# Children’s books about scientists convey demotivating messages

**DOI:** 10.1073/pnas.2612021123

**Published:** 2026-07-21

**Authors:** Jessica R. Gladstone, Gabrielle Applebaum, Andrei Cimpian

**Affiliations:** ^a^ Department of Educational Psychology, University of Illinois, Champaign, IL 61820; ^b^ Department of Psychology, New York University, New York, NY 10003; ^c^ Clinical Psychology Program, Ferkauf Graduate School of Psychology, Yeshiva University, Bronx, NY 10461

**Keywords:** science biographies, role models, content analysis, growth mindset, gender

## Abstract

Biographies of scientists are widely used to spark children’s interest and broaden participation in science. In telling the stories of successful scientists, these books convey implicit “recipes for success”—yet no study has systematically examined whether these recipes align with what motivation research recommends. Here, we provide the first large-scale analysis of motivational messages in children’s science biographies (422 best-selling books, 1,355 protagonists). Four main findings emerged. First, scientific ability was portrayed equally often as fixed and as malleable, a pattern unlikely to support motivation. Second, interest in science was overwhelmingly portrayed as fixed: Most scientists were said to be captivated by science from a young age. Third, scientists in physics, engineering, and computer science were portrayed as more reliant on fixed ability than scientists in other fields. Fourth, biographies of female scientists disproportionately emphasized effort even after adjusting for the obstacles they faced, consistent with stereotypes attributing women’s success to hard work over talent. These findings suggest that well-intentioned efforts to diversify science through role model exposure in children’s science biographies may fall short.

The persistent gender and racial/ethnic disparities in science (1) have prompted efforts to broaden participation, from mentoring programs to media campaigns and informal learning experiences (2, 3). Here, we focus on an important but underexamined element of these efforts: biographies of scientists aimed at children. Although this might seem like a niche genre, children’s science biographies are embedded in the educational ecosystem: Amazon lists over 10,000 such books; widely used elementary curricula include science biographies (4); and many school systems provide educators with access to biographies as a teaching resource (5). Part of their appeal is that these biographies expose children to role models, whom many parents and educators view as important for sparking children’s interest in science (6). Yet biographies of successful scientists do more than introduce children to inspirational people; they also offer implicit “recipes for success” in science, suggesting why and how their protagonists got as far as they did.

Building on prior content analyses of children’s science biographies that have examined demographic representation and portrayals of the nature of science (7, 8), here we provide the first systematic analysis of the motivational content of these books. Such an analysis is important because variations in how scientists’ paths to success are described can shape students’ interest and engagement with science (9). Moreover, if these implicit recipes for success differ systematically based on scientific domain (e.g., physics vs. biology) or based on the gender or race/ethnicity of the featured scientist, they risk reinforcing the inequities that many authors, parents, and educators hope to reduce.

Mindset theory (10) provides an apt framework for evaluating these recipes because it characterizes lay beliefs about the sources of success—precisely what biographies convey when they narrate a scientist’s path. Two dimensions are particularly relevant: how a biography portrays the scientist’s i) ability and ii) interest in science. On each dimension, messages can be more growth-oriented or more fixed. A growth portrayal of ability (that is, describing scientific ability as something developed through practice and mentoring) may make success seem more attainable for children and may thus be motivating (11). In contrast, a fixed portrayal (that is, attributing success to innate, unchangeable talent) implies that only a select few are “born to do science,” a message of exclusivity that can undermine children’s motivation. The same logic applies to interest. A growth portrayal (that is, describing interest in science as developing over time) is likely to make the scientist relatable and their path seem accessible. In contrast, a fixed portrayal (that is, describing the scientist as fascinated by science from a very young age) risks alienating children who have not yet developed a connection to science (12).

The recipes for success conveyed by children’s science biographies may also differ across fields. Some fields are more strongly associated in the public imagination with fixed, innate talent, and these same fields tend to have larger disparities in representation (13). If children’s science biographies echo this pattern, they would be reinforcing the very messages that make some fields seem exclusive and unwelcoming to underrepresented groups. We thus examined whether fixed ability messages differ in prevalence across fields. Specifically, because gender and racial/ethnic disparities are especially pronounced in physics, engineering, and computer science [PECS; (14)], we compared PECS with all other scientific fields.

It also matters whether these recipes differ depending on the scientist’s identity. Here, another kind of message becomes important as well: portrayals of effort as the source of scientific success. Although effort messages are related to growth mindsets, they are not the same (15): A growth mindset message suggests that scientific ability can develop, whereas an effort message suggests that a scientist’s success was the product of (mere) hard work. Although effort is generally positive, portraying success as effort-dependent has negative connotations in science, where effortless talent is valued (13). In addition, a well-documented stereotype holds that women’s and minorities’ achievements are products of hard work rather than talent (16). Thus, if children’s science biographies reproduce this pattern, they could subtly communicate to girls and children of color that scientific success has a higher cost for them compared to others, which may undermine their belonging (17).

To address these questions, we sampled the 500 best-selling children’s science biographies on Amazon. Books were obtained through university and public libraries, as well as through online sources (e.g., YouTube). After exclusions, the final sample consisted of 422 books (*SI Appendix*). These books featured 1,355 scientists (655 unique individuals), 59% male and 41% female; 76% were White and 24% were scientists of color. Roughly half of the protagonists were in PECS.

Two researchers independently coded each protagonist in each book on the presence and strength of fixed and growth mindset messages about ability and interest (0 = *not present*, 1 = *present indirectly*, 2 = *present explicitly*, 3 = *present explicitly more than once*; *SI Appendix*). Interrater reliability was good to excellent [intraclass correlations (ICCs) = 0.73 to 0.90]. Our coding scheme also captured effort portrayals (0 = *not present*, 1 = *present*, 2 = *present more than once*; ICC = 0.86). As expected, effort and growth ability messages were positively correlated but only moderately so (*r* = 0.50), indicating related but distinct constructs.

We analyzed the data using mixed-effects ordinal logistic models. All models adjusted for book length, target age, publication year, and sales rank. We report estimated marginal means, with 95% CIs. Full analytic details are provided in *SI Appendix* and additional results on the Open Science Framework [OSF; (18)].

## Results

**Fig. 1.**
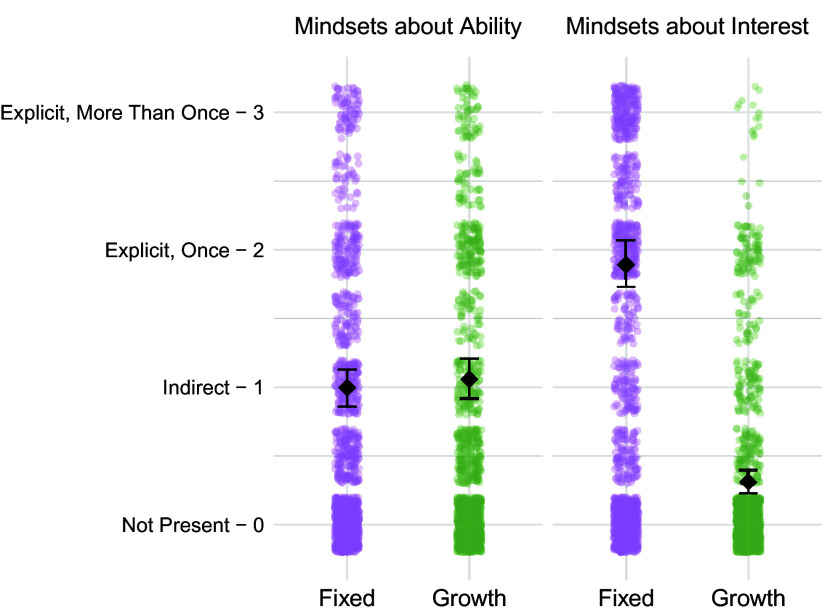
Fixed and growth mindset messages about ability (*Left*) and interest (*Right*). Dots = individual protagonists (jittered). Diamonds = marginal means. Error = 95% CIs. Because scores reflect the average of two coders, data points can fall at half-integer values.

We first examined the overall balance of motivational messages (Fig. 1). Growth and fixed messages about scientists’ ability appeared at similar levels (*M*_fixed_ = 1.00 [0.86, 1.13]; *M*_growth_ = 1.06 [0.92, 1.21]; *b* = 0.14, *OR* = 1.15, *P* = 0.39). Thus, these books were equally likely to discuss scientific ability as inborn and as cultivated. With respect to interest, fixed (vs. growth) messages were overwhelmingly dominant (*M*_fixed_ = 1.89 [1.73, 2.07]; *M*_growth_ = 0.31 [0.23, 0.40]; *b* = −3.41, *OR* = 0.03, *P* < 0.001). Overall, children’s science biographies portray scientific interest as something scientists were essentially born with and scientific ability as just as likely to be innate as developed.

Also notable is the high frequency of “not present” codes (Fig. 1), which indicates that many books do not engage with whether scientific ability and interest develop at all. This is a missed opportunity to convey motivationally helpful messages.

Next, we examined whether mindset messages differed across fields. The success of scientists in PECS (vs. other fields) was described significantly more often as a product of fixed ability (*b* = 0.74, *OR* = 2.10, *P* < 0.001). Growth messages about ability did not show an analogous difference (*b* = 0.25, *OR* = 1.28, *P* = 0.14), and neither did mindset messages about interest (see OSF). Portraying success in PECS as a matter of fixed ability may reinforce the very inequities that parents and educators are trying to combat, particularly for girls and children of color, in some of the fastest-growing areas of science.

**Fig. 2.**
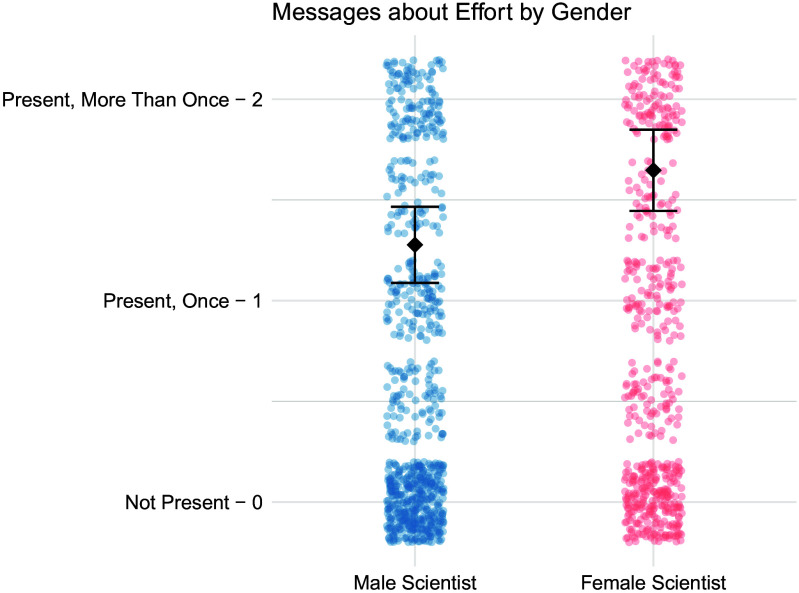
Effort messages, by scientist gender. Dots = individual protagonists (jittered). Diamonds = marginal means. Error = 95% CIs. Because scores reflect the average of two coders, data points can fall at half-integer values.

Finally, we examined whether the messages in children’s science biographies differed by the gender or race/ethnicity of the scientist. A clear gender asymmetry emerged for effort portrayals (Fig. 2): Books about female (vs. male) scientists included substantially more effort messages (*b* = 1.22, *OR* = 3.39, *P* = 0.003). This difference emerged even though we adjusted for domain (PECS vs. non-PECS); thus, it was not merely a byproduct of female scientists being concentrated in particular fields. No analogous difference emerged for race/ethnicity (*b* = 0.01, *OR* = 1.01, *P* = 0.99), or for fixed and growth mindset messages (see OSF). Overall, women’s success seems to be disproportionately framed as the product of hard work, which implies that women’s success in science comes at a higher cost (17).

Perhaps books about female scientists emphasize effort simply because these scientists encountered more barriers, and overcoming barriers involves extra effort. To test this alternative, we added to the preceding model covariates measuring three types of obstacles: failure-related (e.g., failed experiments), social (e.g., unsupportive family), and structural (e.g., sexism) (*SI Appendix*). All three were strong predictors of effort portrayals (*P*s < 0.001), confirming that effort indeed accompanies discussion of obstacles. The gender difference remained significant even after accounting for these obstacles (*b* = 0.76, *OR* = 2.14, *P* = 0.018), indicating that biographies of female scientists emphasize effort above and beyond what can be explained by the obstacles they faced. Finally, the gender difference in effort portrayals was also significant after adjusting for the gender of the books’ authors (*b* = 0.97, *OR* = 2.65, *P* = 0.018), indicating that the asymmetry is not a byproduct of who is writing the books.

## Discussion

Children’s science biographies are a popular way for parents and educators to spark children’s interest in science (4, 5). The evidence here suggests that the implicit recipes for success these books convey are not always well aligned with that goal. Fixed messages about interest predominate, portraying successful scientists as captivated by science from the start rather than as developing their interest over time—a pattern that may alienate children who do not yet see themselves as being interested in science (12). Messages about ability present a more mixed but still potentially problematic picture: Children are just as likely to encounter portrayals of innate ability as portrayals of developed skill. Such mixed fixed/growth messages can be less motivating than no mindset messages at all, in part because fixed framings remain especially salient even when paired with growth framings, leading readers to question whether scientific ability is truly something that can develop (19).

Layered on top of these overall patterns are two asymmetries relevant to inequality. First, biographies of scientists in PECS (vs. other) fields are more likely to portray success in terms of fixed ability, echoing the cultural narratives that make these fields seem especially exclusive (13). Second, biographies of female (vs. male) scientists are more likely to frame success in terms of effort, even after taking into account the obstacles they faced—consistent with a broader cultural narrative in which women’s achievements are due to hard work rather than talent (16, 17).

Do these patterns simply reflect the truth about famous scientists’ lives? Perhaps, for example, most were indeed fascinated by science from a young age. Even if so, authors make choices about which aspects of a scientist’s story to foreground: A scientist’s biography can emphasize their early curiosity or their intellectual journey, and both may be accurate. Our findings suggest that authors’ choices may systematically favor certain narratives (e.g., early-emerging interest, effort for women) in ways that are not well aligned with what motivation science recommends. The goal, then, is not to distort scientists’ stories but to tell them in ways that are both truthful and motivating.

## Materials and Methods

We coded 422 best-selling children’s science biographies for fixed and growth mindset messages about ability and interest, as well as effort portrayals; interrater reliability was good to excellent (ICCs ≥ 0.73). Data were analyzed using mixed-effects ordinal logistic models. Full details are provided in *SI Appendix*.

## Supplementary Material

Appendix 01 (PDF)

## Data Availability

Data, analytic scripts, and additional results have been deposited on OSF (18). All other data are included in the manuscript and/or *SI Appendix*.
