# Supplementary material for: Children’s books about scientists convey demotivating messages
Source: Proc Natl Acad Sci U S A. 2026 Jul 21;123(30):e2612021123. doi: 10.1073/pnas.2612021123 (PMC13415696; doi:10.1073/pnas.2612021123)
Supplement: Supplementary file 1 — Appendix 01 (PDF) [file pnas.2612021123.sapp.pdf]

PNAS

Supporting Information for

Children's Books about Scientists Convey Demotivating Messages

Jessica R. Gladstone[a,b,1], Gabrielle Applebaum[b,c], Andrei Cimpian[b,1]

[a] Department of Educational Psychology, University of Illinois, Champaign, IL 61820

[b] Department of Psychology, New York University, New York, NY 10003

[c] Clinical Psychology Program, Ferkauf Graduate School of Psychology, Yeshiva University, Bronx NY 10461

[1]To whom correspondence may be addressed. Email: jglads2@illinois.edu or andrei.cimpian@nyu.edu.

Author Contributions: J.R.G. and A.C. designed research; J.R.G. and G.A. performed research; J.R.G. analyzed data; and J.R.G. and A.C. wrote the paper.

Competing Interest Statement: The authors declare no competing interests.

Classification: Social Sciences; Psychological and Cognitive Sciences

Keywords: science biographies, role models, children, growth mindset, gender, race, ethnicity, content analysis

This PDF file includes:

Supporting text

Supporting Information Text

Sample

The target sample included the 500 best-selling children's science biographies according to Amazon's U.S. sales rankings as of July 2020. Sampling by sales rank ensured that the books in our dataset reflect the biographies that children are most likely to encounter. In total, out of the top 500 most popular children's science biographies listed on Amazon.com, 422 books were coded. A total of 78 books were excluded because (a) they were not a biography about a scientist, (b) they were written in a language other than English and not available in English, (c) (c) they were part of a gift set, and/or (d) we were unable to be locate a free copy online or via university and local libraries.

The final sample of 422 books featured 1,355 scientist protagonists, of whom 655 were unique individuals (e.g., Isaac Newton appeared in multiple books). Of the 1,355 protagonists, 798 (58.9%) were male and 557 (41.1%) were female; 1,031 (76.1%) were White non-Hispanic and 324 (23.9%) were scientists of color (220 Black, 48 Asian, 28 Middle Eastern or North African, 18 Hispanic or Latinx, 4 Native or Indigenous American or Alaska Native, and 4 multiracial). Because coding of gender and race/ethnicity relied on publicly available information, it should be understood as a coding of publicly presented identities rather than as self-identification on the part of the scientists. Roughly half the protagonists (n = 681) were in physics, engineering, or computer science (PECS), with the remainder (n = 674) in other scientific fields (e.g., biology, medicine).

The 422 books were written by 326 named individual authors and one children's-book imprint ("Baby Professor"), whose books are written by various uncredited writers working under the imprint. Most books had a single author (n = 404, 95.7%); the remaining books (n = 18, 4.3%) had two authors listed (mean number of authors per book = 1.05, range = 1 to 2). Most writers (n = 265, 81.0%) were credited with a single book in our sample (mean number of books per author = 1.35, range = 1 to 8), suggesting that repeated contributions by the same author were limited. Of the 326 named individual authors, 231 (70.9%) were women and 95 (29.1%) were men. Author gender was coded by the first author based primarily on publicly available biographical materials.

Open and Transparent Scientific Practices

The dataset, analytic script, coding scheme, and additional analyses have been deposited on the Open Science Framework (OSF) (https://osf.io/4mqvt/overview?view_only=1530f33b0f744a479914c5eab20e883b) (1).

Coding

Trained researchers coded all books in teams of two, so that each book was independently coded by two researchers. Books featuring multiple protagonists received separate scores for each one. The coding scheme (see below) captured fixed and growth mindset messages about ability and interest, as well as messages about effort and three types of obstacles: failure-related, social, and structural. Whenever a construct was marked as present, the coder recorded a supporting quote to provide a rationale for the assigned code (see below for sample quotes). Coders also recorded each protagonist's demographic information and scientific

domain.

Coders were trained on a separate set of 50 books until each pair achieved an intraclass correlation coefficient (ICC) of at least .75 on every construct. Once this threshold was met, teams began coding the target sample. When the two coders' scores for a given protagonist differed by more than one point, the discrepancy was discussed and resolved; in all other cases, the two scores were averaged to produce the final code. ICCs across the four mindset constructs ranged from .73 to .90, indicating good to excellent reliability (2). Reliability was likewise excellent for the effort construct and the three obstacle constructs (ICCs ranging from .86 to .91).

Analytic Strategy

All analyses were conducted using mixed-effects ordinal logistic regressions (also known as cumulative-link mixed models, or CLMMs) estimated using the *ordinal* package (3) in R (4). All models included crossed random intercepts for book and protagonist to account for the nesting of multiple protagonists within a single book and the same protagonist appearing across multiple books.

To compare the prevalence of growth versus fixed messages, we restructured the data to long format and entered message type (0 = *fixed*, 1 = *growth*) as a fixed effect, with random slopes for message type across books and protagonists. These models also adjusted for scientist gender, scientist race/ethnicity, whether the scientist was in PECS (vs. not), book length (number of pages), target age (lower bound of the suggested age range), publication year, and Amazon sales rank (Box-Cox transformed to address moderate positive skew; skewness reduced from 0.80 to −0.09). All predictors were mean-centered to facilitate interpretation of lower-order coefficients. The continuous covariates were also standardized to facilitate comparison of their coefficients. Regression coefficients were estimated as log odds; we also report odds ratios for interpretability. Estimated marginal means were computed using weighted probabilities across ordinal categories.

To test whether mindset and effort messages differed as a function of domain (PECS vs. not) and the gender or race/ethnicity of the featured scientist, we estimated separate CLMMs for each construct, for a total of five models (fixed ability, growth ability, fixed interest, growth interest, and effort; see OSF). Scientist gender (0 = *man*, 1 = *woman*) and race/ethnicity (0 = *White*, 1 = *scientist of color*) were entered as predictors, along with their interaction. We included random slopes for scientist gender, race/ethnicity, and their interaction to allow these effects to vary across books. PECS (0 = *non-PECS*, 1 = *PECS*) was also included as a fixed effect. Covariates included book length, target age, publication year, and sales rank. In one case (growth ability messages), the model failed to converge with the full random-effects structure. Following best-practice recommendations, we iteratively simplified the random effects; convergence was achieved after removing the random slopes.

To test whether the gender difference in effort portrayals could be explained by the obstacles described in each biography, we re-estimated the effort model after adding three obstacle covariates (failure-related, social, and structural), each coded on a 0–2 ordinal scale (ICCs ≥ .89). In a separate model, we adjusted for the gender composition of each book's author team, ranging from 0 (all male) to 1 (all female), and its interaction with protagonist gender.

Coding Scheme

Construct	Definition	Scale
Fixed Mindset about Ability	Belief that the focal ability is fixed, innate, unchangeable, and inherent.<br><br>“Focal ability” refers to ability in the STEM domain for which the protagonist is well known (e.g., math, chemistry).<br><br>Often, the protagonist’s focal ability in a domain (or domains) has always been there (e.g., since they were a child). If the protagonist discovers their ability for the focal domain later in life, it is because they weren’t exposed to the domain earlier. Their innate ability is instantly discovered when exposed to the focal domain.	0 = Not Present<br>1 = Indirect/Implicit<br>2 = Direct/Explicit<br>3 = Direct/Explicit, Present More Than Once
Growth Mindset about Ability	Belief that the focal ability can be developed and is changeable over time.<br><br>“Focal ability” refers to the ability in the STEM domain for which the protagonist is well known (e.g., math, chemistry).<br><br>The protagonist’s ability in the focal domain (or domains) has not always been there. Instead, their ability grew over time as they learned about the domain and spent time with it. The protagonist’s ability is not instantly discovered when exposed to the focal topic or domain.	0 = Not Present<br>1 = Indirect/Implicit<br>2 = Direct/Explicit<br>3 = Direct/Explicit, Present More Than Once
Fixed Mindset about Interest	Belief that interest in the focal domain is fixed; passion and interest in this domain is innate, unchangeable, and inherent.<br><br>“Focal interest” refers to interest in the STEM domain for which the protagonist is well known (e.g., math, chemistry).<br><br>Often, the protagonist’s interest in the focal domain (or domains) has always been there (e.g., since they were a child). If the protagonist discovers their focal interest later, it is because they weren’t exposed to the focal topic earlier. Their interest or passion was instantly discovered when exposed to that focal topic (“love at first sight”).	0 = Not Present<br>1 = Indirect/Implicit<br>2 = Direct/Explicit<br>3 = Direct/Explicit, Present More Than Once

Growth Mindset about Interest	Belief that interest in the focal domain is malleable; passion and interest in this domain is developed and changeable over time.<br><br>"Focal interest" refers to interest in the STEM domain for which the protagonist is well known (e.g., math, chemistry).<br><br>The protagonist's interest in the focal domain (or domains) has not always been there. Their passion and interest for the focal domain grew over time as they learned about the focal domain and spent time with it. Their interest or passion was not instantly discovered when exposed to the focal domain (no "love at first sight").	0 = Not Present<br>1 = Indirect/Implicit<br>2 = Direct/Explicit<br>3 = Direct/Explicit, Present More Than Once
Effort	The protagonist is said to work hard, put in effort or work, spend a lot of time, etc., in the focal domain.	0 = Not Present<br>1 = Present<br>2 = Present More Than Once[1]
Obstacles: Failure	The protagonist faced failures that made it more difficult for them to succeed.<br><br>Examples of failure obstacles include failing on a project, failing a test, not getting a desired job, etc.	0 = Not Present<br>1 = Present<br>2 = Present More Than Once
Obstacles: Social	The protagonist faced social interactions that made it more difficult for them to succeed.<br><br>Examples of social obstacles include parents not being supportive of chosen career, peers being disparaging, colleagues being harshly critical, etc.	0 = Not Present<br>1 = Present<br>2 = Present More Than Once
Obstacles: Structural	The protagonist faced systemic issues that made it more difficult for them to succeed.<br><br>Examples of structural obstacles include poverty, racism, sexism, discriminatory laws and regulations, politics, etc.	0 = Not Present<br>1 = Present<br>2 = Present More Than Once

Note. In addition to the definitions and scale anchors shown here, coders were provided with hypothetical examples to guide their application of the coding scheme.

[1]The different scales used for mindset versus effort and obstacle constructs reflect a difference in how these constructs typically appear in

children's biographies. Mindset messages about ability and interest were often conveyed indirectly through narrative framing rather than stated outright. Authors rarely wrote that a scientist's ability grew with practice or that their interest developed over time; instead, these mindsets were implied through the structure of the story. Capturing this variation required a scale that could distinguish more indirect from more explicit expressions of a mindset, which is why we used a four-point scale for the mindset constructs. In contrast, effort and obstacles tended to be described more overtly. Because these constructs were rarely implied without also being stated, the indirect-versus-explicit distinction was less informative. For these constructs, a three-point scale distinguishing absence, single mention, and repeated mention was sufficient.

Sample Quotes to Illustrate the Key Variables (Mindsets about Ability and Interest, and Effort)

Book Source	Scientist	Construct	Quote
Little Guides to Great Lives – Stephen Hawking by Isabel Thomas (2019)	Stephen Hawking	Fixed Ability	"At university, Stephen was taught by some of the world's best physicists, but he still found the work easy! He could whiz through complicated calculations without much effort, so he hardly tried at all." (p. 14)
NASA Mathematician Katherine Johnson by Heather E. Schwartz (2017)	Katherine Johnson	Fixed Ability	"But Katherine was born with a natural gift for numbers. Using her talent for math, she excelled in school. And she broke barriers in a career that put her to work as a brilliant mathematician, doing mathematical calculations that would later be done by computers." (p. 5)
Isaac Newton and Physics for Kids: His Life and Ideas with 21 Activities by Kerrie Logan Hollihan (2009)	Isaac Newton	Fixed Ability	"He could see for himself that he was gifted with a remarkable ability to solve difficult problems about vast numbers of subjects." (p. 33)
I am Neil Armstrong by Brad Meltzer (2018)	Neil Armstrong	Growth Ability	"Failure is not an ending. It's an opportunity to learn something new. Whenever you tumble, you must get back up. Every mistake you make teaches you a better way forward." (p. 34)
Starstruck: The Cosmic Journey of Neil deGrasse Tyson by Kathleen Krull and Paul Brewer (2021)	Neil deGrasse Tyson	Growth Ability	"He stretched his brain by inhaling physics, mastering equations, and experimenting" (p. 29)
Margaret and the Moon: How Margaret Hamilton Saved the First Lunar Landing by Dean Robbins (2017)	Margaret Hamilton	Growth Ability	"Margaret began solving harder and harder math problems. It was fun working her way through the steps." (p. 9)
Endurance, Young Readers Edition: My Year in Space and How I Got There by Scott Kelly (2020)	Scott Kelly	Growth Ability	"I may have the will, but I lacked the skills I needed to learn." (p. 20)

Almost Astronauts: 13 Women Who Dared to Dream by Tanya Lee Stone (2009)	Jerrie Cobb	Fixed Interest	"...Jerrie Cobb had been flying airplanes since she was twelve years old. It was in her blood. All she had ever wanted was to keep going higher, faster, farther." (p. 13)
Kid Scientists: True Tales of Childhood from Science Superstars (Kid Legends) by David Stabler (2018)	Vera Rubin	Fixed Interest	"A lifelong fascination with the stars led future astronomer Vera Rubin to search for the answers to these questions, which she first contemplated while gazing through a homemade telescope in her childhood bedroom." (p. 23)
Inventors: Incredible Stories of the World's Most Ingenious Inventions by Robert Winston (2020)	Patricia Bath	Fixed Interest	"Patricia knew from a young age that she wanted to be a doctor" (p. 52)
George Washington Carver: From Slave to Scientist by Janet & Geoff Benge (2001)	George Washington Carver	Growth Interest	"During the course of writing these two books, George developed a deep interest in mycology, the study of fungi such as mushrooms, toadstools, molds, and mildews." (p. 46)
Robo World: The Story of Robot Designer Cynthia Breazeal by Jordan D. Brown (2006)	Cynthia Breazeal	Growth Interest	"In hopes of becoming a physician one day, she considered enrolling in a pre-med program. But then, during her senior year in high school, Cindy developed a greater interest in engineering." (p. 20)
Astronaut Ellen Ochoa (STEM Trailblazer Bios) by Heather E. Schwartz (2017)	Ellen Ochoa	Growth Interest	"Ellen was interested in science, but she did not expect to travel to outer space as an adult. Yet as the world changed around Ellen, so did her dreams… By fifth grade, Ellen thought she might like to be president of the United States one day. But she had many other interests and did not stick with that goal… She was not entirely sure what her career path would be" (pp. 4-7)
Nikola Tesla for Kids: His Life, Ideas, and Inventions, with 21 Activities (For Kids series) by Amy M. O'Quinn (2019)	Nikola Tesla	Effort	"... Nikola nearly drove himself to the point of exhaustion with his course load and hours of study... In his autobiography he wrote 'I regularly started my work at three o'clock in the morning and continued until eleven at night, no Sundays or holidays excepted.'" (p. 17)

Blast Off into Space Like Mae Jemison by Caroline Moss (2020)	Mae Jemison	Effort	“Mae knew that she was going to really have to work; work harder than she had ever worked before, and we all know Mae was used to working very hard.” (p. 32)

SI References

1. J. R. Gladstone, G. Applebaum, A. Cimpian, Data from "Children's Books about Scientists Convey Demotivating Messages." Open Science Framework (OSF). https://osf.io/4mqvt/overview?view_only=1530f33b0f744a479914c5eab20e883b. Deposited 18 May 2026.

2. D. V. Cicchetti, Guidelines, criteria, and rules of thumb for evaluating normed and standardized assessment instruments in psychology. *Psychol. Assess.* **6**, 284–290 (1994).

3. R. H. B. Christensen, ordinal: Regression models for ordinal data. (2026). Deposited 10 January 2026.

4. R Core Team, R: A language and environment for statistical computing. (2023). Deposited 2023.
